# Fungi Fibrinolytic Compound 1 Plays a Core Role in Modulating Fibrinolysis, Altering Plasma Clot Structure, and Promoting Susceptibility to Lysis

**DOI:** 10.3390/pharmaceutics15092320

**Published:** 2023-09-14

**Authors:** Chunli Gao, Bin Bao, Chunling Bao, Wenhui Wu

**Affiliations:** 1 Department of Marine Bio-Pharmacology, College of Food Science and Technology, Shanghai Ocean University, Shanghai 201306, China; chunlgao@163.com (C.G.);; 2 Shanghai Engineering Research Center of Aquatic-Product Processing and Preservation, Shanghai 201306, China; 3 The Sixth People’s Hospital Affiliated, Shanghai Jiao Tong University, Shanghai 201306, China; 4 Putuo Sub-Center of International Joint Research Center for Marine Biological Sciences, Zhongke Road, Putuo District, Zhoushan 316104, China; 5 Marine Biomedical Science and Technology Innovation Platform of Lin-gang Special Area, Lane 218, Haiji Sixth Road, Shanghai 201306, China

**Keywords:** FGFC1, plasma clot, turbidity, clot structure, fibrin lysis, scu-PA, small-molecule natural compound, fibrinolytic activity

## Abstract

Fibrin clot structure and function are major determinants of venous and arterial thromboembolic diseases, as well as the key determinants of the efficiency of clot lysis. Studies have revealed that fungi fibrinolytic compound **1** (FGFC1) is a novel marine pyranisoindolone natural product with fibrinolytic activity. Here, we explore the impacts of FGFC1 on clot structure, lysis, and plasminogen activation in vitro using turbidimetric, enzyme-linked immunosorbent assay, confocal and electron microscopy, urokinase, or plasmin chromogenic substrate. Clots formed in the presence of FGFC1 expressed reduced fibrin polymerization rate and maximum turbidity; however, they did not influence the lag phase of fibrin polymerization. In the absence of scu-PA (single-chain urokinase plasminogen activator), microscopy revealed that FGFC1 increased the number of protofibrils within fibrin fiber and the pore diameter between protofibrils, inducing clots to form a region of thinner and looser networks separated by large pores. The effects of FGFC1 on scu-PA-mediated plasma clot structure were similar to those in the absence of scu-PA. In addition, FGFC1 promoted the lysis of clots and increased the D-dimer concentration in lysate. FGFC1 increased the generation rate of p-nitroaniline in plasma. These results show that FGFC1 has fibrinolytic activity in plasma, leading to interference with the release of fibrinopeptide B to affect lateral aggregation of protofibrils and increase clot susceptibility to fibrinolysis by altering its structure.

## 1. Introduction

The fibrin clot is the final product of the blood coagulation cascade. Fibrin is generated from fibrinogen after thrombin cleavage [1]. Fibrinogen is a 340 kDa glycoprotein composed of six polypeptide chains 2Aα, 2Bβ, and 2 γ [2,3]. The N-termini of all six chains are formed in the E domain, whereas the D domain is composed mainly of the C-termini of the Bβ and γ chains [4]. Fibrinogen is present in the blood at a concentration of 2–4 mg/mL [5,6]. It is a critical component of the coagulation cascade and plays an essential role in platelet aggregation and fibrin clot formation [7]. During coagulation, thrombin catalyzes the cleavage of peptide bonds Arg16-Gly17 and Arg14-Gly15 on the N-termini of the Aα and Bβ chains in the central E-region, respectively, releasing fibrinopeptides A and B to form fibrin monomers [8,9]. Simultaneously, this cleavage also exposes the sites “A” and “B” on one fibrin monomer [3,10]. There is growing evidence that the release of fibrinopeptides A and B contributes to the longitudinal polymerization of fibrin monomers to form protofibrils and aggregate protofibrils laterally to form fibers. “A” site interacts with the “a” site in the γ chain of the distal D region on another fibrin molecule, resulting in the formation of double-stranded protofibrils [11,12]. Knob “B” binds to the complementary hole “b” in the β chain of the distal D region of adjacent molecules to promote lateral aggregation of the protofibrils, forming a three-dimensional network or gel of fibrin clot [13].

The structure and function (such as thickness, permeability, and pore diameter) of clots determine the development and outcomes of various diseases, such as wound healing, surgical complications, fibrinolysis, and heart attack [14]. The early stages of fibrin assembly, branch points, creation, and lateral aggregation of protofibrils essentially shape the fibrin clot’s structure. Therefore, a change in the fibrin fibers’ density, thickness, and stiffness will impact the fibrin clot’s stability and susceptibility to fibrinolysis. Growing evidence indicates that clots display a less permeable and compact fibrin fiber network structure; they seem to be lysis-resistant and more likely to lead to thromboembolism [15]. According to reports, fibrin clots in patients with venous and arterial thromboembolic disease show a denser structure with higher fibrin fiber density and increased resistance to fibrinolysis [16,17].

**Figure 1:**
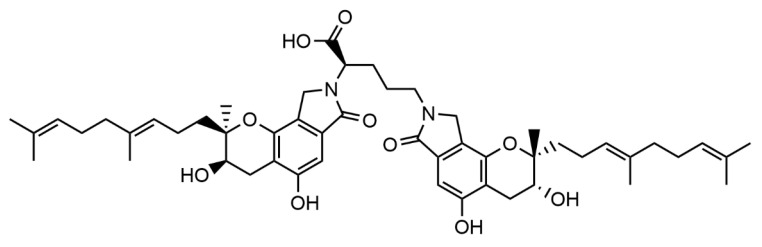
Chemical structure of FGFC1.

Fibrin clots are a new thromboembolic disease risk factor and could represent an essential parameter in patient monitoring [15,18,19]. The structure and formation kinetics of fibrin clots are imperative determinants for their breakdown. They are also necessary for treating, prognosis, and predicting complications in patients with thromboembolic and cardiovascular diseases [20]. Our previous studies showed that the FGFC1[(2,5-bis-[8-(4,8-dimethyl-nona-3,7-dienyl)-5,7-dihydroxy-8-methyl-3-keto-1,2,7,8-tertahydro-6H-pyran[a]isoindol-2-yl]-pentanoic acid)] (Figure 1) with a molecular weight of 869 Da, which can promote plasminogen to plasmin mediated by scu-PA (single-chain urokinase plasminogen activator) [21]. This indicates that FGFC1 is expected to be a new thrombolytic candidate drug [22,23,24]. This study created a plasma fibrinolytic system and incorporated it to assess FGFC1′s fibrinolytic activity in rabbit plasma. By using turbidity, scanning electron microscopy (SEM), laser confocal microscopy, and permeability experiments, we also evaluated the impact of FGFC1 on the fibrin clot structure and lysis.

## 2. Materials and Methods

### 2.1. Materials

FGFC1 was isolated from the metabolites of marine microbial strain *Stachybotrys longispora* FG216 in our laboratory; the purity of FGFC1 was more than 98%. Specifically, the fermentation samples of the target strain were centrifuged after being cultured in a flask shaker at 25 °C and 180 r/min for 7 days. The combined supernatant was extracted with 2-butanone and concentrated in a vacuum at 40 °C. The water layer was adjusted to pH 3.0 with 10% phosphate aqueous solution (*v*/*v*) before salt adjustment to 60% saturation, and then the residue was obtained from the ethyl acetate layer after extraction from the water layer. The isolation was performed on an Inertsil PREP-ODS column (22.5 × 250 mm) at 40 °C. The mobile phase consisted of acetonitrile and 0.1% trifluoroacetic acid with gradient elution at a flow rate of 10 mL/min. Its chemical structure was identified by liquid chromatography-time of flight mass spectrometry (LC-TOFMS) and nuclear magnetic resonance (NMR) [25].

S-2444 chromogenic substrate, scu-PA, and microplate reader were purchased from BioMed (Shanghai, China), Tasly Biopharmaceutical Co., Ltd. (Shanghai, China), and BioTek (Synergy 2, Winooski, VT, USA), respectively. Thrombin, tris (hydroxymethyl)aminomethane (Tris), and fluorescein isothiocyanate isomer I (FITC) were purchased from Sigma-Aldrich (St Louis, MO, USA). The scanning electron microscope was from Hitachi Inc. (SU5000, Tokyo, Japan). The laser scanning confocal microscope was from Leica (Leica TCS SP8, Wetzlar, Germany).

### 2.2. Plasma Preparation

Blood was collected from healthy rabbits anesthetized with chloral hydrate and transferred into an EDTA-K2 anticoagulant tube by cardiac puncture. Platelet-poor plasma was prepared by double centrifugation at 3000× *g* for 10 min at 4 °C and frozen at −80 °C. Before the experiment, the frozen plasma samples were thawed at 4 °C [26].

All studies strictly followed the ARRIVE guidelines and were approved by the institutional animal care and use committees of Shanghai Ocean University.

### 2.3. Turbidity Measurements

Clotting of plasma (final dilution 3:10) was initiated by adding thrombin (0.5 U/mL) and calcium chloride (7.5 mM) in the absence and presence of FGFC1 (0.092–0.92 mM). Turbidity was monitored in 96-well polystyrene plates at 405 nm at 37 °C using a microplate reader every 10 s for 30~60 min. The main parameters, including the lag time, the slope of the polymerization curve, and the maximum absorbance at the plateau, were recorded [15,27]. Note: Final dilution was defined as the ratio of the volume of the plasma stock solution to the total reaction volume.

### 2.4. SEM of Plasma Clots

The structure of plasma clots was analyzed by SEM (SU5000, Tokyo, Japan). Plasma (final dilution 1:6) was incubated with 0.5 U/mL thrombin, 7.5 mM calcium chloride, and FGFC1 (0–0.92 mM) in Tris-HCl buffer at 37 °C for 2 h. Plasma clot samples were washed with Tris-HCl buffer and fixed in 2.5% glutaraldehyde for 2 h. Clot samples were washed three times and freeze-dried after 2 h at room temperature. Clots were sputter coated with 10 nm gold-palladium and examined with a field-emission SEM [28]. ImageJ Version 1.43 (National Institutes of Health) was used to measure the pore diameter and the number of protofibrils within fibrin fiber. Pore diameters were measured at their widest point for all samples. Three representative images of each sample were selected for determination. Note: Final dilution was defined as the ratio of the volume of the plasma stock solution to the total reaction volume.

### 2.5. Laser Confocal Microscopy of Plasma Clots

Plasma (final dilution 1:10), FGFC1 (0–0.92 mm), Tris-HCl buffer, FITC (100 ng/mL), thrombin (0.25 U/mL), and CaCl_2_ (7.5 mM) were added to 12 well chamber slides and incubated at 37 °C for 1–2 h. The reaction mixture was washed three times with Tris-HCl buffer and fixed with 2% glutaraldehyde at room temperature for 2 h. After being washed three times with Tris-HCl buffer, the samples were visualized using a laser confocal microscope (Leica TCS SP8, Germany) on an inverted microscope with a 63× oil immersion objective lens. Note: Final dilution was defined as the ratio of the volume of the plasma stock solution to the total reaction volume.

### 2.6. Plasma Clot Lysis Assay

We used two methods to evaluate the lysis efficiency of clots in the presence of FGFC1. First, clots were formed as described for turbidity by adding plasma (final dilution 3:10) and scu-PA (0.1 nM) before clotting with 0.5 U/mL thrombin and 7.5 mM CaCl_2_. The reaction mixture was measured at 405 nm at 37 °C every 1 min for 6.5 h to record the time of clot lysis. We also detected the level of D-dimer 60 min after scu-PA-mediated clot lysis using enzyme-linked immunosorbent assay (ELISA) in the presence or absence of FGFC1. Additionally, we observed scu-PA-mediated clot structure using a SEM (SU5000, Tokyo, Japan) in the presence and absence of FGFC1. Clots were generated as described above (SEM of plasma clots). ImageJ Version 1.43 (National Institutes of Health) was used to measure the pore diameter between protofibrils within fibrin fiber. Pore diameters were measured at their widest point for all samples. Three representative images of each sample were selected for determination. Note: Final dilution was defined as the ratio of the volume of the plasma stock solution to the total reaction volume.

### 2.7. Determination of Plasminogen Activation in Plasma by FGFC1

Plasma (final dilution 3:10), plasmin chromogenic substrate (0.4 mM), FGFC1 (0, 0.092, 0.184, 0.368, 0.552, 0.736, 0.92, 1.15, 1.38 mM) and Tris-HCl buffer (50 mM, 100 mM NaCl, pH 7.4) were added to 96-well plates. After mixing evenly, the absorbance was detected at 405 nm in the microplate reader at 37 °C, reader every 10 min for 140 min. The absorbance-time curves were plotted and converted into the slope of each curve (Kn). Note: Final dilution was defined as the ratio of the volume of the plasma stock solution to the total reaction volume.

### 2.8. Determination of Plasminogen Activation in Plasma by scu-PA Mediated FGFC1

Plasma (final dilution 3:10), plasmin chromogenic substrate(0.4 mM), FGFC1 (0, 0.092, 0.184, 0.368, 0.552, 0.736, 0.92, 1.15, 1.38 mM), BSA(1%), scu-PA(2 nM) and Tris-HCl buffer(50 mM, 100 mM NaCl, pH 7.4)were added to 96-well plates. After mixing evenly, the absorbance was detected at 405 nm in a microplate reader at 37 °C, read every 10 min for 140 min. The absorbance-time curves were plotted and converted into the slope of each curve (Kn). Note: Final dilution was defined as the ratio of the volume of the plasma stock solution to the total reaction volume.

### 2.9. Determination of scu-PA Activation in Plasma by FGFC1

The reaction mixture contained rabbit plasma (final dilution 3:10), S-2444 (1.6 mM), FGFC1 (0, 0.092, 0.184, 0.368, 0.552, 0.736, 0.92, 1.15, and 1.38 mM), bovine serum albumin (1%), and Tris-HCl buffer (50 mM, 100 mM NaCl, pH 7.4). The absorbance of the mixture system was measured at 405 nm at 37 °C using a microplate reader every 10 min for 140 min. The absorbance-time curves were plotted and converted into the slope of each curve (Kn) to express the fibrinolytic activity of FGFC1. Note: Final dilution was defined as the ratio of the volume of the plasma stock solution to the total reaction volume.

### 2.10. Determination of Plasminogen Activation by scu-PA Mediated FGFC1 In Vitro

Plasminogen (100 nM), FGFC1 (0, 0.4, 0.8, 1.6, 3.2, 6.4, 12.8, 25.6 μM), BSA (1%), plasminogen chromogenic substrate S-2251 (0.17 mM) were added to 96-well plates with or without single-chain urokinase plasminogen activator (scu-PA, 1 nM) in Tris-Hcl buffer (50 mM, 100 mM NaCl, pH 7.4). The absorbance of the mixture system was measured at 405 nm at 37 °C using a microplate reader every 5 min for 155–255 min. The absorbance-time curves were plotted and converted into the slope of each curve (Kn).

### 2.11. Statistical Analysis

Statistical analyses were performed using Prism 8 (GraphPad) using the *t*-test. Two-sided *p* < 0.05 were regarded as statistically significant. Unless otherwise stated, the data are presented as mean ± standard deviation (SD).

## 3. Results

### 3.1. FGFC1 Effect on Clot Turbidity

**Figure 2:**
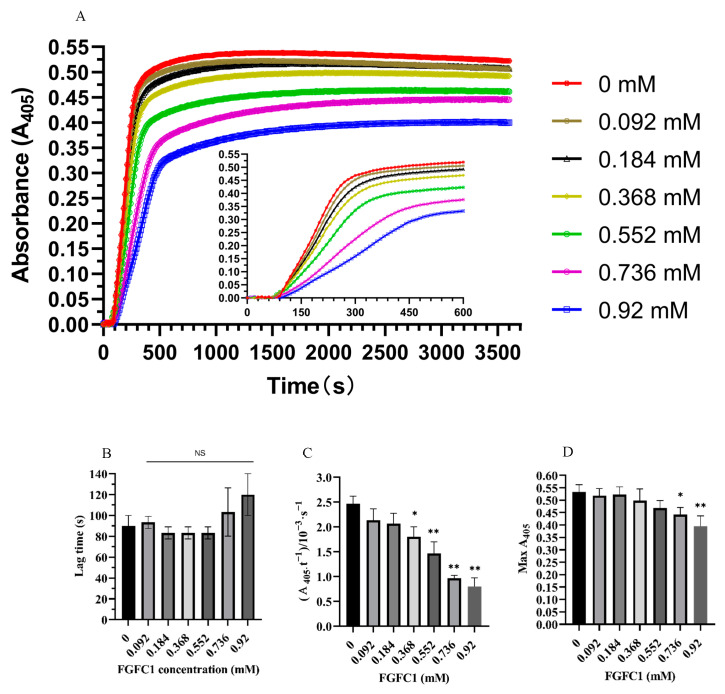
Effect of FGFC1 (0–0.92 mM) on the kinetics of fibrin clot formation. The turbidity was monitored at 405 nm every 10 s for 30 min, and the results were expressed as mean values of three experiments. (**A**) Effect of FGFC1 (0–0.92 mM) on dynamic clot turbidity. Clots were made with the addition of thrombin (0.5 U/mL) and 7.5 mM calcium chloride in the absence and presence of FGFC1 (0.092–0.92 mM) (**B**) Effect of FGFC1 on the lag time of fibrin polymerization, defined as the time from initiation of plasma clot formation to when absorbance increases to 0.015 from baseline. (**C**) The linear slope between the initial baseline and the mid-point of the maximum absorbance at the plateau. (**D**) Maximum absorbance of plasma clot at 405 nm. The mean ± SD of three experiments is shown. NS, not significant, * *p* < 0.05, ** *p* < 0.01, compared with vehicle (absence of FGFC1).

Turbidity kinetic analysis was performed to determine whether FGFC1 could regulate the formation of plasma clots by measuring the absorbance of the reaction mixture at 405 nm using a microplate reader. The results showed that FGFC1 prolonged the lag time of clot formation in a dose-dependent manner, but there was no significant difference compared with the absence of FGFC1 (Figure 2A,B).

FGFC1 (0–0.92 mM) also reduced the rate of fibrin polymerization in a dose-dependent manner. When FGFC1 concentrations were 0.368, 0.552, 0.736, and 0.92 mM, the fibrin polymerization rates were (1.8 ± 0.2) × 10^−3^ s^−1^, (1.47 ± 0.23) × 10^−3^ s^−1^, (0.97 ± 0.058) × 10^−3^ s^−1^, and (0.8 ± 0.17) × 10^−3^ s^−1^, respectively (*p* < 0.05, *p* < 0.01) (Figure 2A–C). Additionally, FGFC1 also significantly reduced the maximum turbidity of plasma clots (0.44 ± 0.028 (*p* < 0.05) and 0.39 ± 0.042 (*p* < 0.01) for 0.736 and 0.92 mM FGFC1, respectively) (Figure 2D). These data show that FGFC1 can directly hinder the formation of plasma clots and may affect the structure of the plasma fibrin network.

### 3.2. Impacts of FGFC1 on Fibrin Structure by Scanning Electron Microscopy

**Figure 3:**
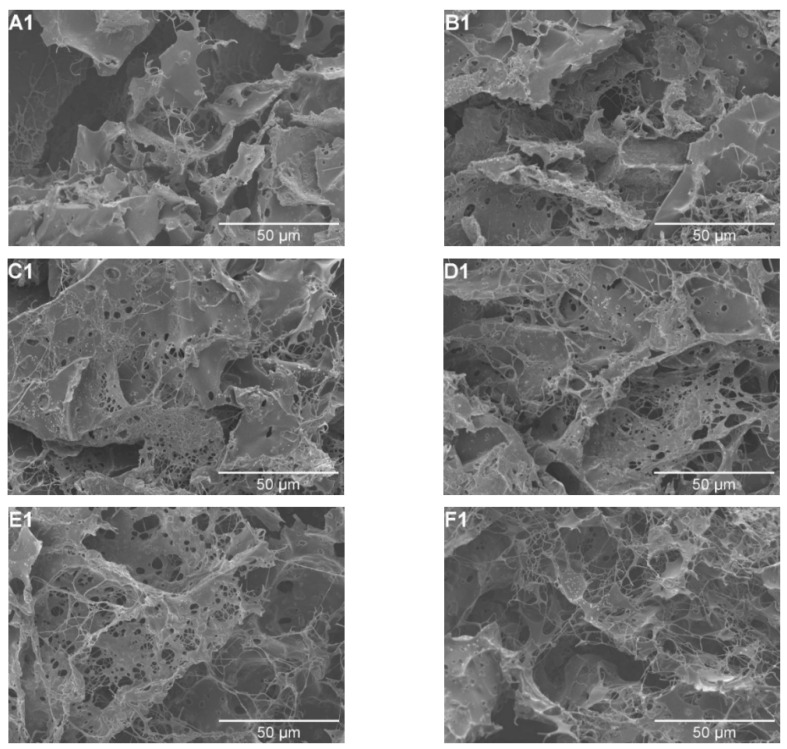

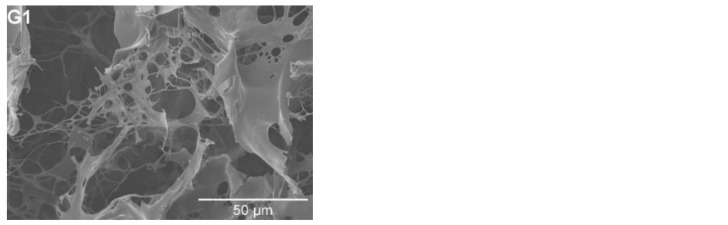
Effect of FGFC1 (0–0.92 mM) on plasma clot structure. Plasma clots were prepared by incubating plasma (final dilution 1:6) with thrombin (0.5 U/mL) and CaCl_2_ (7.5 mM) in the absence (**A1**) and the presence (**B1**–**G1**) of FGFC1 (0.092–0.92 mM). Scale bars represent 50 μm. The concentrations of FGFC1 in Figure (**A1**–**G1**) were 0, 0.092, 0.184, 0.368, 0.552, 0.736, and 0.92 mM, respectively.

We used SEM and laser confocal microscopy to study the effect of FGFC1 on clot structure and showed that FGFC1 changes clot structure in a concentration-dependent manner. Without FGFC1, a clot forms a relatively homogenous fiber or block structure. In contrast, clots prepared in the presence of FGFC1 showed an abnormal, partially conglutinated, entangled, and loose fibrin network that is interspaced with large pores (Figure 3A1–G1).

**Figure 4:**
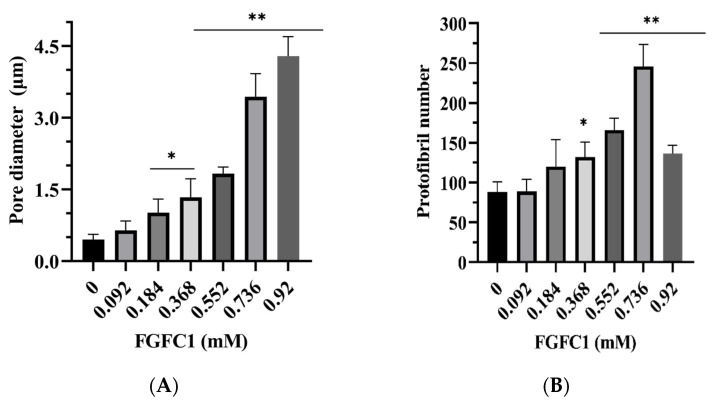
FGFC1-induced changes in plasma clot ultrastructure (clots were analyzed by scanning electron micrographs). The pore diameter between protofibrils (**A**). (**B**) Number of protofibrils within fibrin fiber. Results are expressed as a mean ± SD. *n* = 3. * *p* < 0.05, ** *p* < 0.01, compared with vehicle (absence of FGFC1).

Figure 3A1–G1 illustrates how FGFC1 alters the ultrastructure of fibrin by increasing the pore diameter between protofibrils and increasing the number of protofibrils. The pore diameters of protofibrils increased from 0.45 ± 0.1 µm in the control group to 4.29 ± 0.4 µm (*p* < 0.05, *p* < 0.01) in the 0.92 mM FGFC1 group. When the concentration of FGFC1 was 0.736 mM, the number of protofibrils increased by about 1.78-fold compared with that in the absence of FGFC1 (246 ± 28 vs. 88 ± 13). However, when FGFC1 concentration was 0.92 mM, the number of protofibrils was less than that at 0.736 mM (Figure 4A,B), indicating that FGFC1 prevented the aggregation of fibrin. Thus, FGFC1 can significantly affect the structure of plasma clots.

### 3.3. Impacts of FGFC1 on Fibrin Structure by Confocal Laser Microscopy

**Figure 5:**
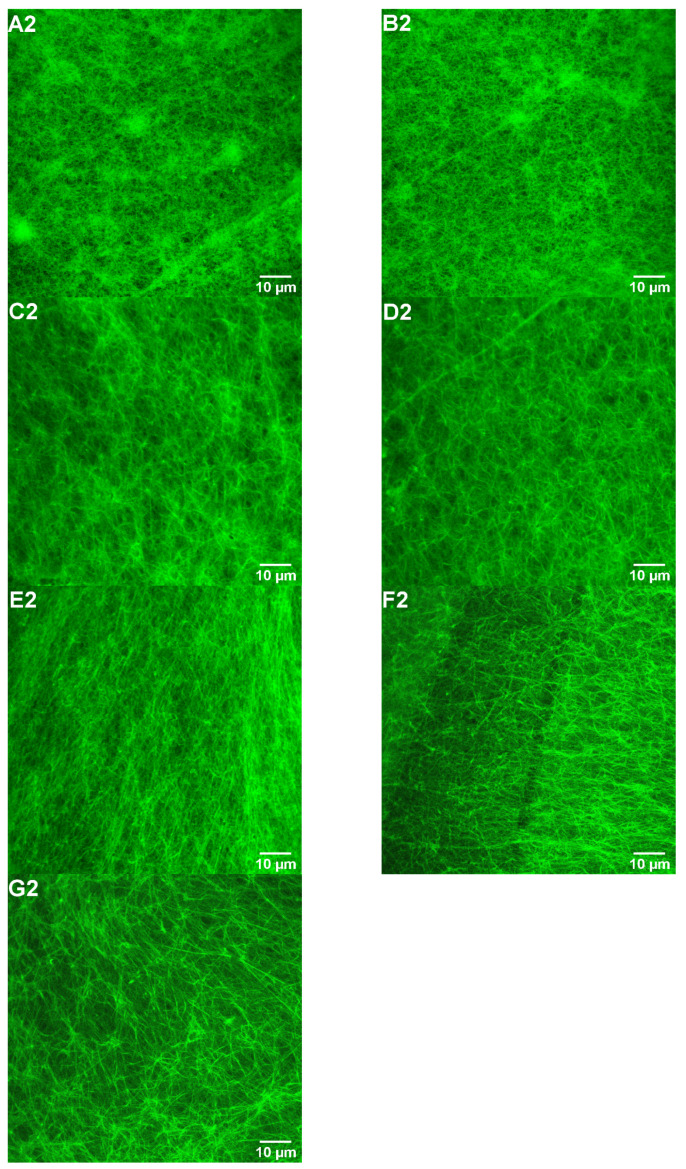
Effect of FGFC1 (0–0.92 mM) on plasma clot structure. Plasma clots were prepared by incubating plasma (final dilution 1:10) with thrombin (0.25 U/mL) and CaCl_2_ (7.5 mM) in the absence (**A2**) and the presence (**B2**–**G2**) of FGFC1 (0.092–0.92 mM). (**A2**–**G2**) Plasma clots were analyzed by laser scanning confocal microscopy after fluorescein isothiocyanate staining. The scale bar represents 10 μm. Note: There were some shortcomings in conducting experiments without using fluorophore-conjugated fibrinogen.

Confocal laser microscopy revealed that FGFC1 altered fibrin fiber binding, entanglement, and aggregation in plasma clots to create a fibrin network with low fiber aggregation and entanglement regions. Among them, the high concentrations of FGFC1 reveal clear differences in fibrin network structure compared with the control group, and its fibrin formed a fiber region with less tight fiber arrangement and branchpoint (Figure 5A2–G2).

### 3.4. Effects of FGFC1 on Fibrinolysis In Vitro

#### 3.4.1. FGFC1 Promotes Fibrinolysis

**Figure 6:**
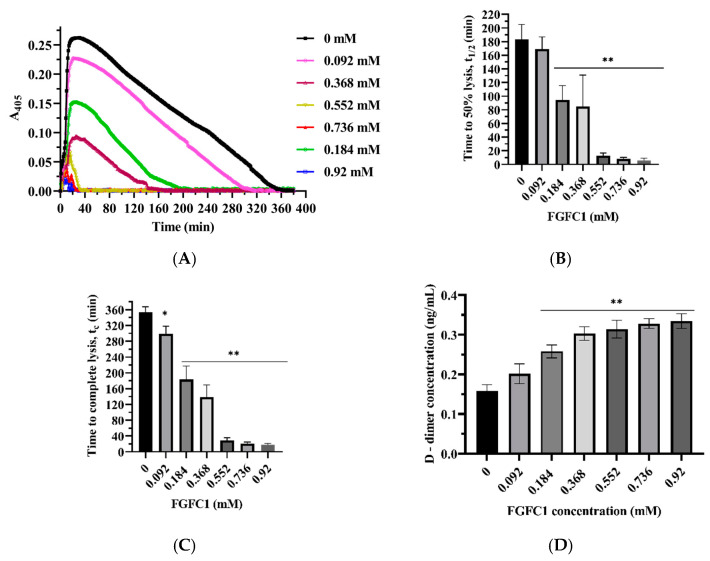
FGFC1 (0.092–0.92 mM) promotes scu-PA-mediated lysis of clots. (**A**) Plasma, scu-PA, and FGFC1 (0–0.92 mM), thrombin (0.5 U/mL), and CaCl_2_ (7.5 mM) were incubated at 37 °C to form clots, and dynamic clot turbidity was measured at 405 nm. Results were expressed as mean values of three experiments. (**B**) Defined as the time between the midpoint from clear to MT and the half-maximal A405 value (t1/2). (**C**) The time from initiation of clotting to complete dissolution of clots (tc). (**D**) ELISA was used to detect the D-dimer level after scu-PA-mediated fibrinolysis of plasma clots for 1 h in the absence and presence of FGFC1. Results expressed as a mean ± SD.* *p* < 0.05, ** *p* < 0.01, compared with vehicle (absence of FGFC1).

Next, we investigated whether FGFC1 modulates clots’ susceptibility to lysis. Changes in clot structure and turbidity dynamics greatly influence fibrinolysis. Based on these changes, we evaluated the effect of FGFC1 on scu-PA-induced lysis of clots. Scu-PA-mediated fibrinolysis was promoted when FGFC1 was added to plasma and scu-PA during clot formation (Figure 6).

As the concentration of FGFC1 increases, the time to 50% and complete lysis of clots were significantly dose-dependent (*p* < 0.05, *p* < 0.01) (Figure 6A–C). For example, when the concentration of FGFC1 was from 0.092 to 0.92 mM, the time (t1/2) from initiation of clotting to the midpoint of the maximum turbidity and the half-maximal A405 value decreased from 169 ± 17.6 min to 6 ± 3 min, which reduced by approximately 98% (Figure 6B). These results suggest that FGFC1 has an excellent promoting effect on scu-PA-mediated plasma clot lysis.

#### 3.4.2. Effects of FGFC1 on D-Dimer Formation

D-dimer is a marker of fibrin formation and fibrinolysis, closely related to thrombosis. It is frequently used in clinical practice to rule out deep-vein and venous thrombosis. The concentration of D-dimer was measured using enzyme-linked immunosorbent assay (ELISA) with or without incubation with FGFC1 and scu-PA (Figure 6D). Results indicated that D-dimer levels were significantly enhanced in the presence of increasing FGFC1 concentrations. When the concentrations of FGFC1 were 0.184, 0.368, 0.552, 0.736, and 0.92 mM, concentrations of D-dimer were significantly increased (*p* < 0.01) compared with those without FGFC1 (0.158 ± 0.016), which were 0.258 ± 0.016, 0.303 ± 0.017, 0.314 ± 0.023, 0.328 ± 0.012, and 0.334 ± 0.019 ng/mL, respectively. The results suggest that FGFC1 has a good fibrinolytic effect in vitro.

#### 3.4.3. FGFC1 Influences Fibrin Structure Mediated by scu-PA

**Figure 7:**
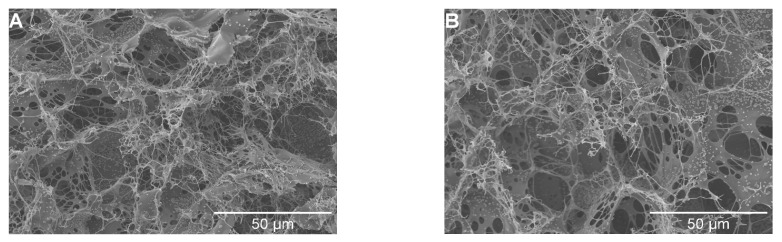

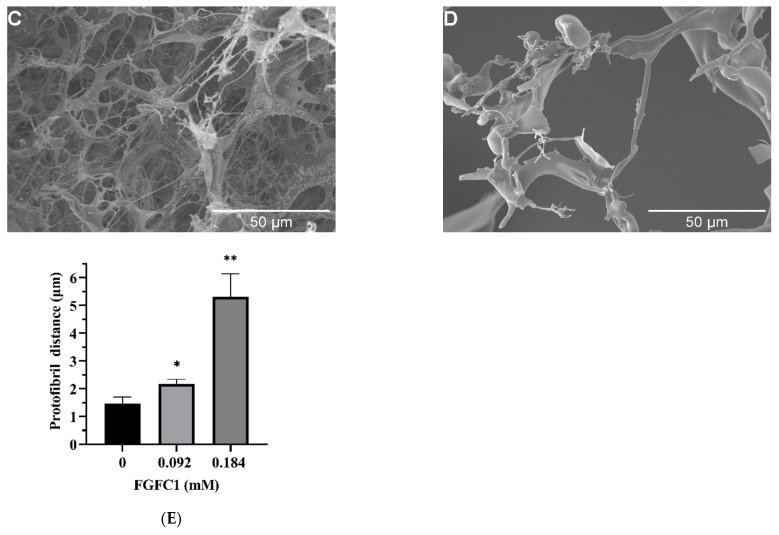
Effect of FGFC1 (0–0.368 mM) on scu-PA-mediated plasma clot structure. Plasma clots were formed by incubating plasma (final dilution 3:10) with thrombin (0.5 U/mL) and CaCl_2_ (7.5 mM) in the absence (**A**) and the presence (**B**–**D**) of FGFC1 (0.092–0.368 mM). Distance between protofibrils (**E**). Results are expressed as a mean ± SD. n = 3. * *p* < 0.05, ** *p* < 0.01, compared with vehicle (absence of FGFC1). Representative SEM images and scale bars (50 μm) of fibrin clots are shown (**A**–**D**).

The effect of FGFC1 (0–0.92 mM) on the scu-PA-mediated (0.1 nM) clot structure was analyzed by SEM. As seen in Figure 7, FGFC1 still affects the fibrin clot structure in a concentration-dependent manner. The change in FGFC1 on the scu-PA-mediated (0.1 nM) clot structure appears similar to that without scu-PA (Figure 3A1–G1). Still, a lower concentration of FGFC1 could cause clots to form a network structure with tightly wrapped and cross-linked fibers in the presence of scu-PA. With the increased concentrations of FGFC1, the pore diameter of fibers gradually increased to form a loose network structure with thinner fibers and fewer branching points, resulting in more sensitivity to lysis (Figure 7).

With the concentration of FGFC1 increasing from 0 to 0.184 mM, branch points of the fiber network decreased together with an increase in the pore diameters of the protofibrils from 1.45 ± 0.24 µm in the control group to 2.16 ± 0.17 μm (0.092 mM) and 5.3 ± 0.84 μm (0.184 mM), showing increases of approximately 0.5-fold and 2.7-fold, respectively (*p* < 0.05, *p* < 0.01) (Figure 7E). When the concentrations were 0.184 and 0.368 mM, the clot was almost completely dissolved, resulting in only a thicker clot, indicating that the clots were gradually lysed with the increase in FGFC1 concentration. These results indicate that after the addition of FGFC1, the efficiency of plasma clot dissolution mediated by scu-PA is higher, but only from the change of plasma clot microstructure in the presence and absence of scu-PA (Figure 3 and Figure 7), FGFC1 can significantly change the plasma clot structure, and the changing trend is consistent. The results further suggest that FGFC1 can change the structure of plasma clots and increase their sensitivity to scu-PA dissolving clots.

### 3.5. Effect of FGFC1 on Plasminogen Activation in Plasma

**Figure 8:**
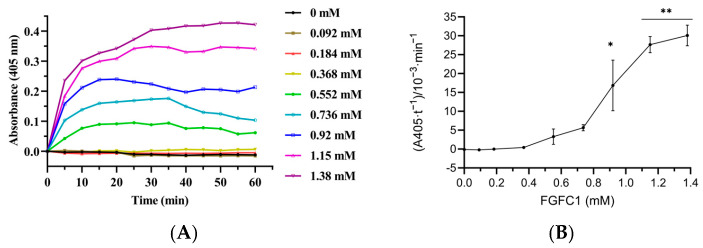
Effect of FGFC1 on the activation of plasminogen to plasmin in plasma. (**A**) Absorbance-time curve. (**B**) Plasmin generation is presented as rate of chromogenic substrate hydrolysis. Results are expressed as a mean ± SD. * *p* < 0.05, ** *p* < 0.01, compared with vehicle (absence of FGFC1).

On the basis of the above research, the effect of FGFC1 on plasminogen activation in plasma without scu-PA was investigated by using the plasmin chromogenic substrate method (S2251). The results are shown in Figure 8. When the concentration of FGFC1 was less than 0.368 mM, there was no significant difference in the color reaction rate compared with the control group, indicating that the plasminogen in the plasma was not activated into plasmin. When the concentration of FGFC1 increased from 0.552 mM to 1.38 mM, FGFC1 increased the chromogenic reaction rate in a concentration-dependent manner, which increased by about 8.2 times ((30.1 ± 2.8) × 10^−3^ min^−1^ vs. (3.3 ± 2.1) × 10^−3^ min^−1^) (*p* < 0.01). In addition, the chromogenic reaction rate was very fast, reaching the maximum reaction rate at about 10 min, indicating that with the increase in FGFC1 concentration, more plasminogen was activated into plasmin. The results showed that high concentrations of FGFC1 also had a strong ability to activate plasminogen in plasma without adding scu-PA, thereby promoting the degradation of plasma clots. At the same time, this may explain why FGFC1 significantly reduced the aggregation rate and maximum absorbance value and changed the structure of clots in the clot turbidity experiment in plasma without scu-PA.

### 3.6. The Effect of scu-PA-Mediated FGFC1 on Plasminogen Activation in Plasma

**Figure 9:**
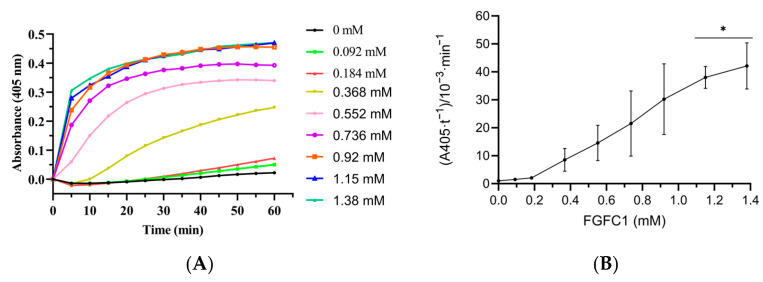
Effect of FGFC1 on the activation of plasminogen to plasmin in plasma in the presence of scu-PA. (**A**) Absorbance-time curve. (**B**) Plasmin generation is presented as the rate of chromogenic substrate hydrolysis. Results are expressed as a mean ± SD. * *p* < 0.05, compared with vehicle (absence of FGFC1).

After the addition of scu-PA (2 nM) to plasma, the activation of plasminogen by FGFC1 is shown in Figure 9. The rate of plasmin production also showed a concentration-dependent trend with FGFC1. Compared with the reaction system without scu-PA (Figure 8), the lower concentration of FGFC1 can promote an increase in absorbance value at 405 nm. When the concentration of FGFC1 was 1.38 mM, the reaction rate increased by about 27 times compared with 0.092 mM ((42.1 ± 8.3) × 10^−3^ min^−1^ vs. (1.5 ± 0.3) × 10^−3^ min^−1^) (*p* < 0.05). These indicated that the addition of scu-PA to the plasma can significantly improve the ability of FGFC1 to induce plasminogen into plasmin.

### 3.7. Fibrinolytic Activity of FGFC1 in Plasma

**Figure 10:**
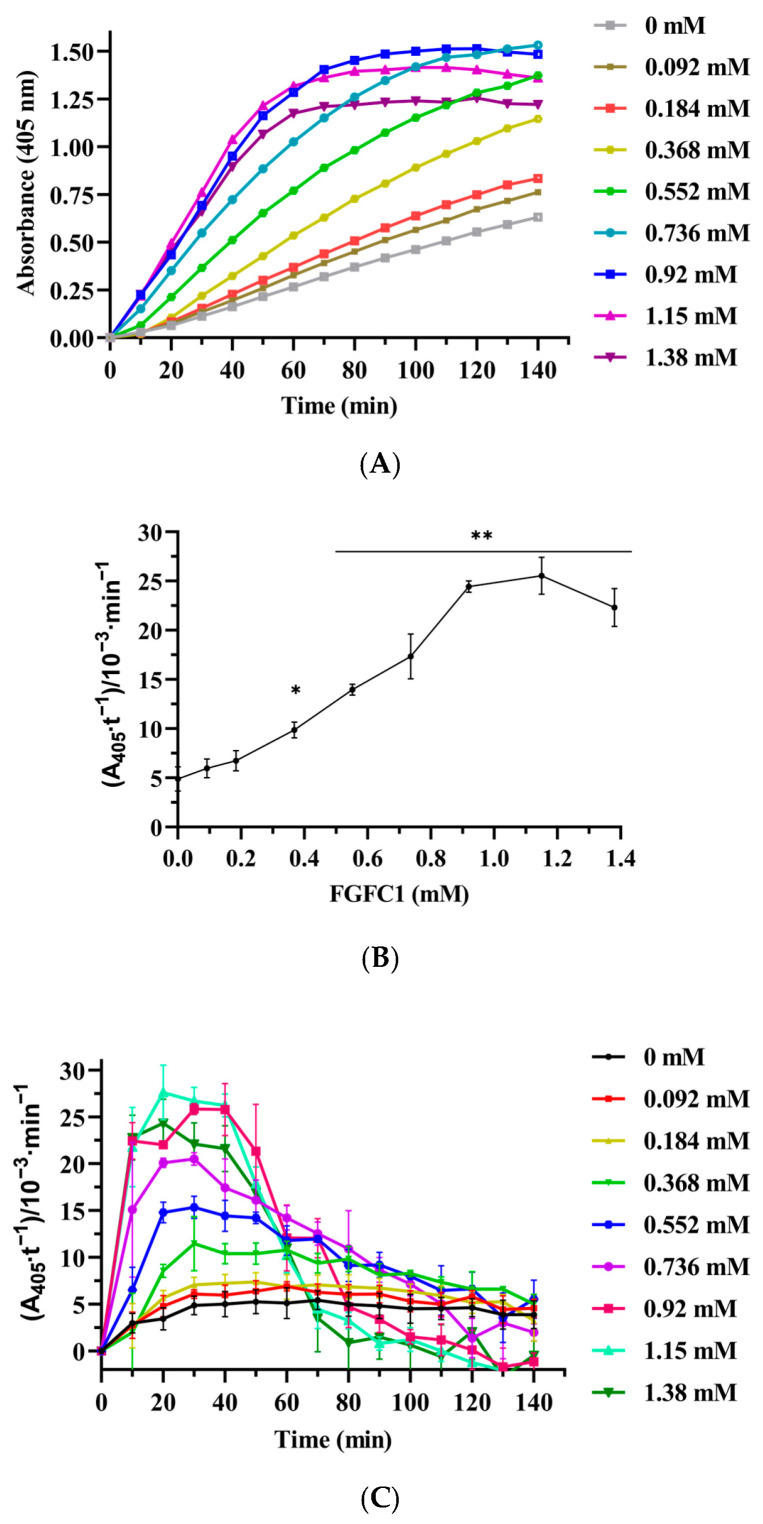
FGFC1 (0–1.38 mM) promotes the conversion of S-2444 to p-NA in plasma. (**A**) The absorbance-time curve of the reaction system containing FGFC1. The reaction was incubated at 37 °C and measured every 10 min for 140 min. (**B**) FGFC1-induced changes in the values of Kn (the slope of each curve) in the reaction mixture system. Results expressed as a mean ± SD.* *p* < 0.05, ** *p* < 0.01, compared with vehicle (absence of FGFC1). (**C**) Reaction rates of FGFC1 (0–1.38 mM) at different times (0–140 min).

The substrate S-2444 can be hydrolyzed to p-nitroaniline (p-NA) catalyzed by urokinase, fibrinolytic enzyme, and kallikrein, showing a light yellow color. In this reaction mixture system, the effects on the color reaction of S-2444 were investigated by adding FGFC1 (0–1.38 mM). The hydrolysis of S-2444 to p-NA followed the Michaelis–Menten kinetics [29] when the chromogenic substrate S-2444 was in excess, the reaction rate of p-NA formation was proportional to both the concentration of FGFC1 and the A405 values of the reaction system measured at 405 nm. The findings showed that the linear range of the color reaction dynamic curve increased with decreasing FGFC1 concentration. The coloring rate of the response system peaked between 20 min and 40 min within a specified concentration range of FGFC1. When the concentrations were 1.15 and 1.38 mM, the reaction rates reached their maximum at 20 min; after 40 min, the color reaction rate showed a downward trend (Figure 10A–C).

The effect of FGFC1 (0–1.38 mM) on the reaction rate presented two trends, including the enhancement and weakening of promoting effects (Figure 10A–B). The reaction rate at 1.15 mM was the highest, close to the rate at 0.92 mM. The reaction rate for 1.38 mM FGFC1 showed a weakening promotion trend compared with 1.15 mM. FGFC1 (0–1.15 mM) concentration-dependently affected the reaction rate. From 0 to 1.15 mM, the reaction rate increased from (4.90 ± 1.23) × 10^−3^ min^−1^ to (25.53 ± 1.89) × 10^−3^ min^−1^ (*p* < 0.01), which increased by about 4.2-fold (Figure 10B). These results indicated that FGFC1 increased the conversion of plasminogen to plasmin in plasma, which further converted sc-uPA into uPA and then hydrolyzed its chromogenic substrate S-2444 to produce p-NA. It is also implied that FGFC1 can promote the dissolution of intravascular thrombus.

### 3.8. Effect of FGFC1 on Plasminogen Activation In Vitro

**Figure 11:**
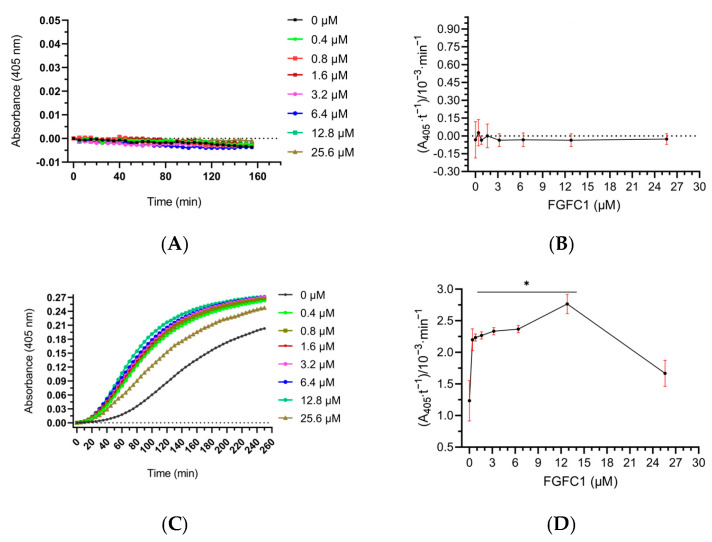
Effect of FGFC1 at different concentrations mediated by scu-PA on the conversion of plasminogen to plasmin. The reaction was incubated at 37 °C and measured every 5 min for 155–255 min. (**A**) The kinetic curve of plasminogen conversion to plasmin and (**B**) the reaction rate diagram mediated by FGFC1 in the absence of scu-PA. (**C**) Kinetic curve of plasminogen conversion to plasmin and (**D**) the reaction rate diagram mediated by FGFC1 in the presence of scu-PA. Results expressed as a mean ± SD.* *p* < 0.05, compared with vehicle (absence of FGFC1).

To study whether FGFC1 can activate plasminogen in vitro, we simulated the human body environment to construct a reaction mixture system containing plasminogen and used the plasmin chromogenic substrate method to investigate the effect of FGFC1 on plasminogen activation. It can be seen from Figure 11 that when there was no scu-PA in the system, the color reaction rate of S2251 did not change with the increase in FGFC1 concentration and reaction time. It is suggested that plasminogen is not activated into plasmin in this reaction system. However, in the mixed system containing scu-PA, when the concentration of FGFC1 was lower than 12.8 μM, the color reaction rate of the system was significantly higher than that of the control group and was concentration-dependent with FGFC1. When the concentration of FGFC1 was 25.6 μM, the activation rate of plasminogen showed a decreasing trend, which was not statistically significant compared with the control group, indicating that the production of plasminogen was inhibited. These results suggest that FGFC1 cannot activate plasminogen in the absence of scu-PA.

## 4. Discussion

FGFC1, a marine pyranoindolone alkaloid small molecule compound, was discovered in the metabolites of the S. longispora FG216. It was found that FGFC1 can promote the dissolution of FITC-fibrin mediated by plasminogen and scu-PA in vitro [21,30]. In vivo, FGFC1 (10 mg/kg) can effectively dissolve most of the pulmonary thrombosis of Wistar rats induced by extrinsic FITC-fibrin [24,31]. Additionally, FGFC1 shows linear pharmacokinetics and distributes rapidly in most tissues except the brain in Wistar rats [22]. In this study, we assessed the impact of FGFC1 on the structure and function of plasma clots using turbidimetry, clot lysis assays, scanning electron microscopy, and confocal microscopy. The results showed that FGFC1 significantly reduced the aggregation rate and maximum turbidity of plasma clots. The clot structure is formed by interspersing large pores in the filament winding area in the presence of FGFC1. FGFC1 can effectively promote the dissolution of plasma clots mediated by scu-PA. FGFC1 can promote the activation of plasminogen in plasma with or without scu-PA. However, comparatively speaking, the addition of scu-PA can significantly improve the ability of FGFC1 to activate plasminogen, and lower concentrations of FGFC1 can promote the hydrolysis of S-2444 to pNA in plasma. The results showed that FGFC1 had good fibrinolytic activity in plasma, which could change the structure of plasma clots and quickly dissolve plasma clots.

Turbidimetry is performed to monitor the development of fibrin clots. During fibrin polymerization in solution, fibrinopeptide A released rapidly. Our turbidity data show that FGFC1 did not affect the lag phase of fibrin polymerization, indicating that fibrinopeptide A was released and protofibrils were formed typically [27,32]. The release rate of fibrinopeptide B is slower than that of fibrinopeptide A and reaches the maximum when fibrin is included, promoting lateral aggregation when protofibrils reach a sufficient length [33]. In the turbidity experiment, the release of fibrinopeptide B is reflected by the rapid increase in turbidity [2,34]. There are many factors and analysis conditions affecting turbidity. In purified systems, maximum turbidity (MT) is directly related to the average fiber cross-sectional area. However, in plasma samples, MT is associated with fibrinogen concentration, fiber diameter, other clot characteristics, and cardiovascular disease. At the same time, these characteristics also affect coagulation and fibrinolysis [8,35]. In the presence of FGFC1, fibrin’s maximum absorbance and polymerization rate were considerably reduced, indicating that FGFC1 may change the fiber diameter. The analysis of plasma clot structure by scanning electron microscopy and confocal microscopy showed that no significant changes in fiber diameter were observed in the presence of FGFC1. These differences may be related to the amount of rabbit plasma used in this study. Structural analysis of clots by SEM and confocal microscopy showed that the fibers’ diameter was not significantly changed in the presence of FGFC1, which may be related to the amount of plasma used in this study. However, we observed a fibrin network structure with more prominent pores in tightly entangled fiber clusters. These results may explain the decrease in the polymerization rate and maximum absorbance of fibrin with FGFC1 observed in the turbidity experiment.

The ability of fibers to migrate during the early stages of polymerization has been demonstrated using microscopy. The time at which the fibers move and the beginning of scaffold construction correspond to the lag period in the turbidity curve. The longitudinal growth and branching point formation of fibers complete at approximately 60% of MT, while lateral growth of fibers continues until network formation is fully completed [36]. Microscopy showed that a fiber network structure with large pores was formed after incubation of plasma with FGFC1. Furthermore, our data on fibrin clot structure showed a significant increase in the pore diameter of the clot and thinner and less densely packed fibers in the presence of FGFC1, resulting in more sensitivity to lysis. These structural analyses and turbidity data on the clots show that FGFC1 might interfere with the release of fibrinopeptide B to affect the lateral aggregation of fibrin. It also confirmed the potential beneficial value of FGFC1 in thrombolysis.

A fibrin clot’s network topology influences its sensitivity to lysis [37,38]. Turbidity change at the microscopic level and fiber dissolving rate at the macroscopic level are used to measure fibrinolysis [39]. Based on the above studies, we further studied whether FGFC1 could regulate the sensitivity of fibrin to lysis through a clot lysis experiment in vitro. The results showed that scu-PA-mediated lysis was promoted when FGFC1 was added during clot formation, and the time to attain 50% lysis was increased. Moreover, we measured D-dimer levels in plasma clot lysate using an ELISA after incubating the reaction mixture for 60 min. The results indicated that the concentration of D-dimer was increased in the presence of increasing concentrations of FGFC1. These findings suggest that the clot formed by thrombin and calcium chloride is more resistant to lysis when FGFC1 is absent. Additionally, the effect of FGFC1 on the structure of plasma clots mediated by scu-PA is consistent with the absence of scu-PA. Fibrin clots structure in the absence of FGFC1 shows areas of more tightly knit fibers that are interspaced with smaller pores in contrast to less dense and the number of branch points in FGFC1 clots. It suggests that FGFC1 delays clot formation and is more sensitive to lysis. In the presence of scu-PA, only a lower concentration of FGFC1 can significantly change the structure of plasma clots, indicating that the effect of a high concentration of FGFC1 on plasma clots’ structure is similar to that of scu-PA. This further indicates that FGFC1 may play a role in the fibrinolytic pathway by affecting the structure of the clot.

**Figure 12:**
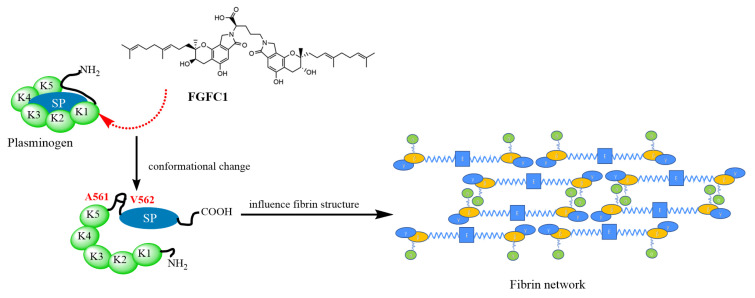
The role of FGFC1 in plasma was preliminarily speculated.

The activation of the plasminogen activator triggers the fibrinolytic system in the plasma. Common activators include tPA released by the endothelium and uPA produced by various cell types [40,41]. However, uPA activity was rarely detected in normal plasma [42]. It has been reported that scu-PA is present in plasma in an inert form and cannot directly activate plasminogen [42]. In contrast, scu-PA encounters plasminogen with morphological changes on the surface of the thrombus, which can significantly increase the fibrinolytic activity of scu-PA, resulting in the conversion of plasminogen to plasmin [35,43]. Plasmin further activates scu-PA to tc-uPA, containing two disulfide bonds, hydrolyzing S-2444 to p-NA [30,35]. Scu-PA is also a plasminogen activator that does not bind to plasminogen activator inhibitor-1 (PAI-1) in plasma [44,45]. Based on these, the plasma reaction mixture system and a fibrinolytic system simulating the human body environment for monitoring the enzymatic kinetics of the S-2251 and S-2444 chromogenic reaction were constructed to detect the fibrinolytic activity of FGFC1. The results showed that the production of plasmin was not detected in the fibrinolytic system containing plasminogen, FGFC1 (0.4–12.8 µM) without scu-PA in vitro. However, FGFC1 (0.4–12.8 µM) significantly promoted plasminogen activation into plasmin in the presence of scu-PA. In addition, the change of absorbance value under a low concentration of FGFC1 (0.092–0.368 mM) was not detected when scu-PA was not added, while the high concentration of FGFC1 (0.552–1.38 mM) could significantly promote the activation of plasminogen in plasma, but the required concentration of FGFC1 was tens or even hundreds of times higher than that required FGFC1 (0.4–12.8 μM) in the fibrinolytic system constructed in vitro. When scu-PA was added to the plasma, a low concentration of FGFC1(0.092 mM) could promote the chromogenic reaction rate of S2251 and S-2444. In summary, we initially speculated that FGFC1 in plasma changes the conformation of plasminogen to be activated by the original scu-PA in the plasma into plasmin so that the plasma clot or fibrin can be quickly dissolved, resulting in changes in the structure of plasma clots and reducing the polymerization rate and maximum absorbance of plasma clots in turbidity experiments (Figure 12). However, the choice to work on the plasma of healthy rabbits may have potential influencing factors; therefore, our results need further verification in patients with thromboembolism.

## 5. Conclusions

In conclusion, this study shows that FGFC1 has good fibrinolytic activity in rabbit plasma. Furthermore, FGFC1 can reduce plasma clots’ aggregation rate and MT, enhancing lysis sensitivity. The structural characteristics of fibrin clots are significant in preventing and treating thrombosis. Our findings offer a theoretical foundation for additional investigation into the mechanism by which FGFC1 affects plasma clots and serve as a guide for future clinical research on FGFC1 in thrombotic diseases. Overall, this study provides a fundamental research basis for further research on the effects of FGFC1 on fibrinolysis.

## Data Availability

Not applicable.
